# Comprehensive analysis the prognostic and immune characteristics of mitochondrial transport-related gene SFXN1 in lung adenocarcinoma

**DOI:** 10.1186/s12885-023-11646-z

**Published:** 2024-01-17

**Authors:** Wenting Liu, Qingwu Du, Ting Mei, Jingya Wang, Dingzhi Huang, Tingting Qin

**Affiliations:** Department of Thoracic Oncology, Tianjin Key Laboratory of Cancer Prevention and Therapy, Tianjin Medical University Cancer Institute and Hospital, National Clinical Research Center for Cancer, Tianjin’s Clinical Research Center for Cancer, Tianjin, China

**Keywords:** SFXN1, Lung adenocarcinoma, Biomarker, Prognosis, Tumor immune microenvironment

## Abstract

**Background:**

Mitochondria, which serve as the fundamental organelle for cellular energy and metabolism, are closely linked to the growth and survival of cancer cells. This study aims to identify and assess Sideroflexin1 (SFXN1), an unprecedented mitochondrial gene, as a potential prognostic biomarker for lung adenocarcinoma (LUAD).

**Methods:**

The mRNA and protein levels of SFXN1 were investigated based on the Cancer Genome Atlas (TCGA) LUAD dataset, and then validated by real-time quantitative PCR, Western Blotting and immunohistochemistry from our clinical samples. The clinical correlation and prognostic value were evaluated by the TCGA cohort and verified via our clinical dataset (*n* = 90). The somatic mutation, drug sensitivity data, immune cell infiltration and single-cell RNA sequencing data of SFXN1 were analyzed through public databases.

**Results:**

SFXN1 was markedly upregulated at both mRNA and protein levels in LUAD, and high expression of SFXN1 were correlated with larger tumor size, positive lymph node metastasis, and advanced clinical stage. Furthermore, SFXN1 upregulation was significantly associated with poor clinical prognosis. SFXN1 co-expressed genes were also analyzed, which were mainly involved in the cell cycle, central carbon metabolism, DNA repair, and the HIF-1α signaling pathway. Additionally, SFXN1 expression correlated with the expression of multiple immunomodulators, which act to regulate the tumor immune microenvironment. Results also demonstrated an association between SFXN1 expression and increased immune cell infiltration, such as activated CD8 + T cells, natural killer cells (NKs), activated dendritic cells (DCs), and macrophages. LUAD patients with high SFXN1 expression exhibited heightened sensitivity to multiple chemotherapies and targeted drugs and predicted a poor response to immunotherapy. SFXN1 represented an independent prognostic marker for LUAD patients with an improved prognostic value for overall survival when combined with clinical stage information.

**Conclusions:**

SFXN1 is frequently upregulated in LUAD and has a significant impact on the tumor immune environment. Our study uncovers the potential of SFXN1 as a prognostic biomarker and as a novel target for intervention in LUAD.

**Supplementary Information:**

The online version contains supplementary material available at 10.1186/s12885-023-11646-z.

## Background

 Lung cancer continues to be the foremost cause of cancer-related morbidity across the globe, as evidenced by the recent data presented by Siegel et al. [1]. Adenocarcinoma, squamous cell carcinoma, and large cell carcinoma are subtypes of non-small cell lung cancer (NSCLC), which accounts for more than 85% of lung cancer cases [2]. Adenocarcinoma is the most commonly diagnosed subtype of NSCLC, and its incidence rate is on the rise [3]. The significant advances in genetic diagnosis and molecular targeted therapy have greatly improved the effectiveness of treatment, particularly for LUAD. The advent of EGFR-TKIs has led to the emergence of precision medicine based on tumor molecular alteration profile as opposed to tumor histology or anatomy [4, 5]. However, the 5-year overall survival rate remains below 20%, largely due to resistance to targeted drugs and inadequate identification of additional mutant driver genes [6]. As a result, identifying additional essential genes and prognostic biomarkers is crucial at this time to improve early diagnosis, customized treatment plans, and overall prognosis.

Abnormal energy metabolism is a pivotal feature of cancer [7]. Mitochondria, by regulating cellular energy and metabolism, play a vital role in the growth and survival of cancer cells [8]. Furthermore, mitochondria have been recently identified as key regulators of immune cells, controlling both innate and adaptive immune responses through their role in the establishment and maintenance of immune cell phenotypes [9]. Although mitochondrial dysfunction is a shared characteristic of most cancers, the underlying mechanisms of these phenomena remain unresolved [10]. While some drugs targeting mitochondrial metabolism have shown promising effects in treating cancer, their clinical application is currently limited [11]. The identification of genetic alterations in mitochondria is critical for a comprehensive understanding of tumor metabolism. Additionally, this knowledge could be utilized in developing mitochondrial metabolism-based therapies for lung cancer [10, 12].

The SFXN family is a conserved group of mitochondrial proteins in humans [13]. It is composed of SFXN1, also known as the tricarboxylate carrier protein [14], SFXN2, SFXN3, SFXN4 and SFXN5. SFXN2 is primarily expressed in the kidney, SFXN3 in the retina, SFXN4 in the pancreas, and SFXN5 in the brain, while SFXN1 is ubiquitously expressed as a multi-pass protein on the inner membrane of mitochondria [15]. Recently, Nora Kory and colleagues verified that SFXN1 serves as a mitochondrial serine transporter in the process of one-carbon metabolism and is highly expressed in many cancers [15]. Studies have shown that SFXN1 was upregulated in LUAD and may promote the proliferation and metastasis through mTOR signaling pathway [16, 17]. However, to the best of our knowledge, there are relatively few studies on the research of SFXN1 mutational expression profile and its relationship with drug sensitivity and immune infiltration in LUAD.

To elucidate the possible relevance between the SFXN family and LUAD, we conducted an integrative analysis of the SFXN family, particularly SFXN1, through various cancer-related databases such as TCGA, Gene Expression Omnibus (GEO), and Clinical Proteomic Tumor Analysis Consortium (CPTAC), and deeply investigated the relationship between SFXN1 expression and various immune cell infiltrations and clinical drug sensitivity. We validated the prognostic value of SFXN1 in LUAD using our clinical data with the hope of providing useful insights into the mitochondrial genetic etiology of lung cancer.

## Materials and methods

### Samples and cell lines

In the training set, we downloaded transcriptome expression data and corresponding clinical samples from the TCGA database (https://portal.gdc.cancer.gov/), including 492 LUAD and 59 adjacent normal tissue samples. Patients included in the study needed to have complete gene expression data, follow-up information, and TNM staging. The gene expression data values were converted into log2(TPM + 1) format. LUAD patients were divided into high and low groups by the median expression of SFXN1 mRNA. In the validation set, 20 adjacent non-tumor samples and 90 LUAD samples that underwent surgery between December 2012 and February 2014 were obtained from Tianjin Cancer Institute and Hospital, which was approved by the ethics committee of the Tianjin Medical University Cancer Institute and Hospital (bc2023085) and adhered to the ethical guidelines of the Helsinki Declaration. Termination time of follow-up of our clinical data was May 15, 2023. Cell lines used in this study, including BEAS-2B, NCI-H1299, A549, and NCI-H1975 were provided by American Type Culture Collection (ATCC) cell bank.

### Gene expression analysis by real-time quantitative PCR (qRT-PCR)

The total RNA was extracted using the Trizol reagent following the product protocol (Invitrogen, No.15596026). The reverse transcription reaction and qPCR were performed based on our previous study [18]. SFXN1 primers followed the 5’ to 3’ direction: ACC AGT CCT TCA ATG CCG TCG T (forward) and GAG TCC TAG AGC TGT TGC TAC G (reverse) (CAT#: HP214538). We used GAPDH as the reference primer (Forward: GTC TCC TCT GAC TTC AAC AGC G; Reverse: ACC ACC CTG TTG CTG TAG CCA A (CAT#: HP205798). The experiment was repeated three times. Other primers are shown in the Supplementary Table 1.

### Western blotting (WB)

The protein extraction and western blotting processes were performed as described in our previous publication [19]. Protein concentration was quantified with BCA protein assay (Solarbio, PC0020). Subsequently, 30–50 µg of proteins were separated by 10% SDS-polyacrylamide gel electrophoresis (PAGE) and then transferred onto polyvinylidene fluoride (PVDF) membranes. After blocking the membranes with blocking buffer (EpiZyme, Cat#: PS108P, Shanghai, China) for 30 min, the primary antibodies were added and the membranes were incubated overnight at 4 °C. The membranes were then washed three times (10 min each) with Tris-buffered saline containing 0.1% Tween-20 (TBST) (EpiZyme Biotech, Cat#: PS103S). Horseradish peroxidase (HRP)-conjugated secondary antibodies (CST) were incubated with the membranes for 1 h at room temperature, followed by an additional three washes with TBST. Protein signals were detected by chemiluminescence using Super ECL Detection Reagent (Cat.No. WBKLS0500, Millipore, USA). The primary antibodies were listed below: β-Actin (1:1000 dilution, CST Cat#3700), SFXN1 (1:1000 dilution, Proteintech Cat#12296-1-AP). Western blot stripping buffer (P0025) was purchased from Beyotime Biotechnology (Shanghai, China). Note: To detect protein expression on the same membrane, under the premise of ensuring the integrity of the target proteins, we hybridized with two different antibodies successively on the same blotting membrane, using the stripping buffer in the process.

### Immunohistochemistry (IHC)

The detailed IHC procedures and SFXN1 staining score criteria were followed by our previous study [18]. Antigen retrieval was performed with pressure cooking in tris-EDTA (pH = 9.0). We used a rabbit polyclonal antibody against SFXN1 (Proteintech Cat#12296-1-AP; RRID: AB 2,185,814) at a dilution of 1:200. The IHC score no more than seven was regarded as low expression and vice versa for high expression.

### Cell counting Kit-8 assay (CCK8)

To assess the inhibitory effect of drugs on cell proliferation, A549 cells infected with control lentivirus and SFXN1-overexprssion lentivirus were seeded into 96-well plates at a density of 2000 cells per well. At 24, 48, 72 h after the cells were seeded, CCK-8 reagent (Zeta, France) was mixed with the cells for 2 h incubation at 37 °C in the dark. The absorbance value was measured at 450 nm with Microplate reader.

### Expression landscape and prognostic value of SFXN family genes

We utilized multiple bioinformatic databases to investigate the expression and prognostic value of SFXN1 and other SFXN family genes across different cancer types. The differential expression of SFXN1 in pan-cancerous tissues and normal tissues was examined using the ULCAN database (https://ualcan.path.uab.edu/index.html) [20]. The prognostic value of SFXN family genes in LUAD patients was evaluated by univariate Cox regression analysis. Differences in SFXN family genes expression between LUAD and adjacent normal tissues were obtained from the GEPIA database (http://gepia.cancer-pku.cn/) [21]. The correlation between SFXN1 mRNA expression and protein expression was analyzed by the cProSite database (https://cprosite.ccr.cancer.gov/) and protein expression differences of SFXN1 in LUAD and normal tissue were accessed via the CPTAC database (https://hupo.org/Clinical-Proteome-Tumor-Analysis-Consortium-(CPTAC)). The prognostic value of SFXN1 at the pan-carcinoma level was evaluated using the GEPIA2.0 database (http://gepia2.cancer-pku.cn/#index) [22]. Correlations between SFXN1 expression and clinicopathological features in LUAD patients were analyzed using the ANOVA or Wilcoxon test followed by visualization through the “ggplot2” package.

### Functional enrichment analysis

To explore the biological function of SFXN1, a total of 296 differentially expressed genes in LUAD patients with low and high SFXN1 expression using the “limma” package, applying a screening criterion of |LogFC|>0.7. Meanwhile, the gene ontology (GO) analysis was performed to validate the role of SFXN1 in LUAD and then the Gene Set Enrichment Analysis (GSEA) was used to identify enriched pathways. The first eight enriched pathways are shown in the figure. Additionally, protein-protein interaction (PPI) analysis was performed to identify co-expressed genes of SFXN1, using a correlation index greater than 0.4 as the criterion, and the PPI networks was generated from the STRING database (https://cn.string-db.org/) [23, 24]. The most correlated subnetworks were identified using the Mcode plugin of Cytoscape and their biological functions were visualized through the Cluego plugin.

### Somatic mutation analysis

Somatic mutation data for LUAD patients were obtained from the TCGA database. The correlation between SFXN1 expression and tumor mutational burden (TMB) value was analyzed by pearson correlation test. TMB was subsequently calculated for each LUAD patient using the “maftools” package, which was also utilized to generate mutant waterfall plots and enrichment pathways for both low and high SFXN1 expression groups.

### Immune infiltration analysis

The “ESTIMATE” package was utilized to calculate the estimate score, immunity score, stroma score, and tumor purity for every LUAD sample. To investigate the impact of SFXN1 on the composition of tumor-infiltrating immune cells, we utilized the “GSVA” package along with the single-sample GSEA (ssGSEA) algorithm to evaluate the immune scores of 28 immune cell types. A Wilcoxon test was then employed to assess the correlation between SFXN1 and immune checkpoints by comparing low and high SFXN1 expression groups. To evaluate the differentiation between tumor immune dysfunction and exclusion (TIDE) scores, we uploaded the normalized gene expression matrix to the TIDE website (http://tide.dfci.harvard.edu) and the Wilcoxon test was utilized to compare the estimates of different SFXN1 expression groups. Futhermore, we analyzed the correlation between SFXN1 expression and immune microenvironment factors through TISIDB [25] (http://cis.hku.hk/TISIDB/index.php), including lymphocytes, immunostimulators, immunoinhibitors, MHC molecules, chemokines and chemokine receptors, which is an integrated database for tumor–immune system interactions.

### Drug sensitivity analysis

The chemotherapy response of LUAD patients from the TCGA database was evaluated based on the drug sensitivity data from the Cancer Genome Project (CGP) database. The half-maximal inhibitory concentrations (IC50) values of 251 antitumor drugs were estimated using the “pRRophetic” package, which utilized the gene expression matrix from individual LUAD patient. Comparative analysis of drug sensitivity was performed on the high and low SFXN1 expression groups. Additionally, the correlation coefficient was calculated by the Spearman correlation test between drug sensitivity and SFXN1 expression.

### Analysis of SFXN1 expression in single-cell level of NSCLC

The multidimensional features of NSCLC-infiltrating lymphocytes were identified using the Peking University database (http://lung.cancer-pku.cn/index.php). This database contained advanced deep single-cell RNA sequencing data and comprehensive T-cell receptor (TCR) information. The single-cell level data regarding SFXN1-related functional status was obtained from the Cancer SEA database (http://biocc.hrbmu.edu.cn/CancerSEA/).

### Prognostic analysis

The Kaplan–Meier (KM) analysis was conducted based on the survival time in the low and high group of SFXN1 expression (training set and validation set). Univariate and multivariate Cox regression analysis was performed to examine the correlation between survival time, clinical prognostic indicators and SFXN1 expression using the “survival” package. Nomograms were constructed based on the independent factors of Cox multivariate analyses in the TCGA database by “rms” package. The concordance index (C-index) and calibration were assessed to effectively measure the performance of constructed nomograms. The “SurvivalROC” package was applied to generate receiver operating characteristic (ROC) curves to assess predictive performance.

### Statistical analysis

R programming language (Version 4.0.4) and GraphPad Prism 9 were utilized for statistical analysis. A significance level of *P* < 0.05 was applied with bilateral detection for all statistical analyses.

## Results

### Expression and prognostic value of SFXN1 across cancers

**Fig. 1:**
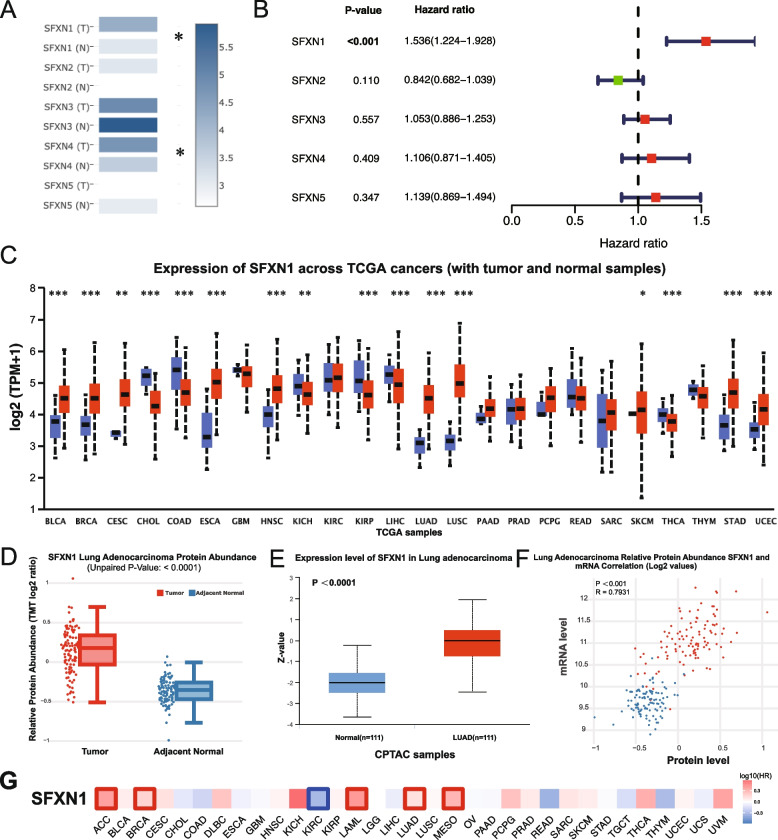
SFXN1 expression levels and prognostic value in different cancers, specifically in LUAD. **A** SFXN family genes expression in LUAD (T) and normal lung tissues (N). **B** Univariate Cox regression of SFXN family genes for OS in LUAD. **C** SFXN1 expression levels in tumors (red) and normal tissues (blue) from TCGA. **D** SFXN1 protein abundance in TCGA LUAD and healthy tissue. **E** SFXN1 protein expression in normal and LUAD from CPTAC database. **F** Correlation between mRNA level and the protein abundance of SFXN1. **G** Correlation between the SFXN1 expression and OS in pan-cancers based on the TCGA data

Our analysis of the GEPIA database revealed that SFXN4, another member of the SFXN family, was overexpressed in lung adenocarcinomas (Fig. 1A). Subsequently, univariate Cox regression of the five genes of SFXN family using 495 LUAD patients within the TCGA database revealed that only high expression level of SFXN1 were associated with poor patient prognosis within LUAD (Fig. 1B). Analysis of the TCGA database suggested that the expression of SFXN1, a member of the SFXN family of genes, was significantly upregulated in several types of cancer, including lung cancer, hepatocellular carcinoma, and breast cancer, as compared to paracancerous tissues (Fig. 1C). The clinical characteristics of the LUAD patients analyzed within the TCGA database are presented in Table 1. TCGA and CPTAC database analysis indicated that the abundance of SFXN1 protein was significantly higher in LUAD patients as compared to normal lung tissues (Fig. 1D, E). We observed a clear correlation between the abundance of SFXN1 protein and its mRNA expression levels, which was able to effectively distinguish between LUAD and paraneoplastic tissues (*R* = 0.7931, *P* < 0.001) (Fig. 1F). Lastly, we assessed the potential prognostic value of SFXN1 expression in a range of cancer types within the TCGA database. Our results indicated that high expression levels of SFXN1 were associated with improved prognosis in kidney renal clear cell carcinoma (KIRC), but with poor prognosis in several other cancers, including LUAD, adrenocortical carcinoma (ACC), breast invasive carcinoma (BRCA), acute myeloid leukemia (LAML), and mesothelioma (MESO) (Fig. 1G).

**Table 1:** The clinical characteristics of LUAD patients from the TCGA database

Clinical variables	*N* = 492	%
**Age (years)**		
Mean (SD)	65.4 (10.0)	–
Median (Min, Max)	66 (33, 88)	–
< 60	134	27.23
≥ 60	358	72.76
**Gender**		
Female	264	53.66
Male	228	46.34
**Race**		
White	385	78.25
Non-White	59	12.00
Unknown	48	9.75
**Smoking history**		
Non-smoker	71	14.43
Smoker	407	82.72
Unknown	14	2.85
**Clinical stage**		
I	266	54.07
II	120	24.39
III	80	16.26
IV	26	5.28
**Tumor size (cm)**		
T1 (≤ 3)	169	34.35
T2 (3–5)	258	52.44
T3 (5–7)	44	8.94
T4 (> 7)	18	3.66
Unknown	3	6.10
**Lymph node metastasis**		
Negative	317	64.43
Positive	165	33.54
Unknown	10	2.03
**Distant metastasis**		
Negative	328	66.67
Positive	25	5.08
Unknown	139	28.25

### Relationship between SFXN1 and clinicopathologic features of LUAD patients

**Fig. 2:**
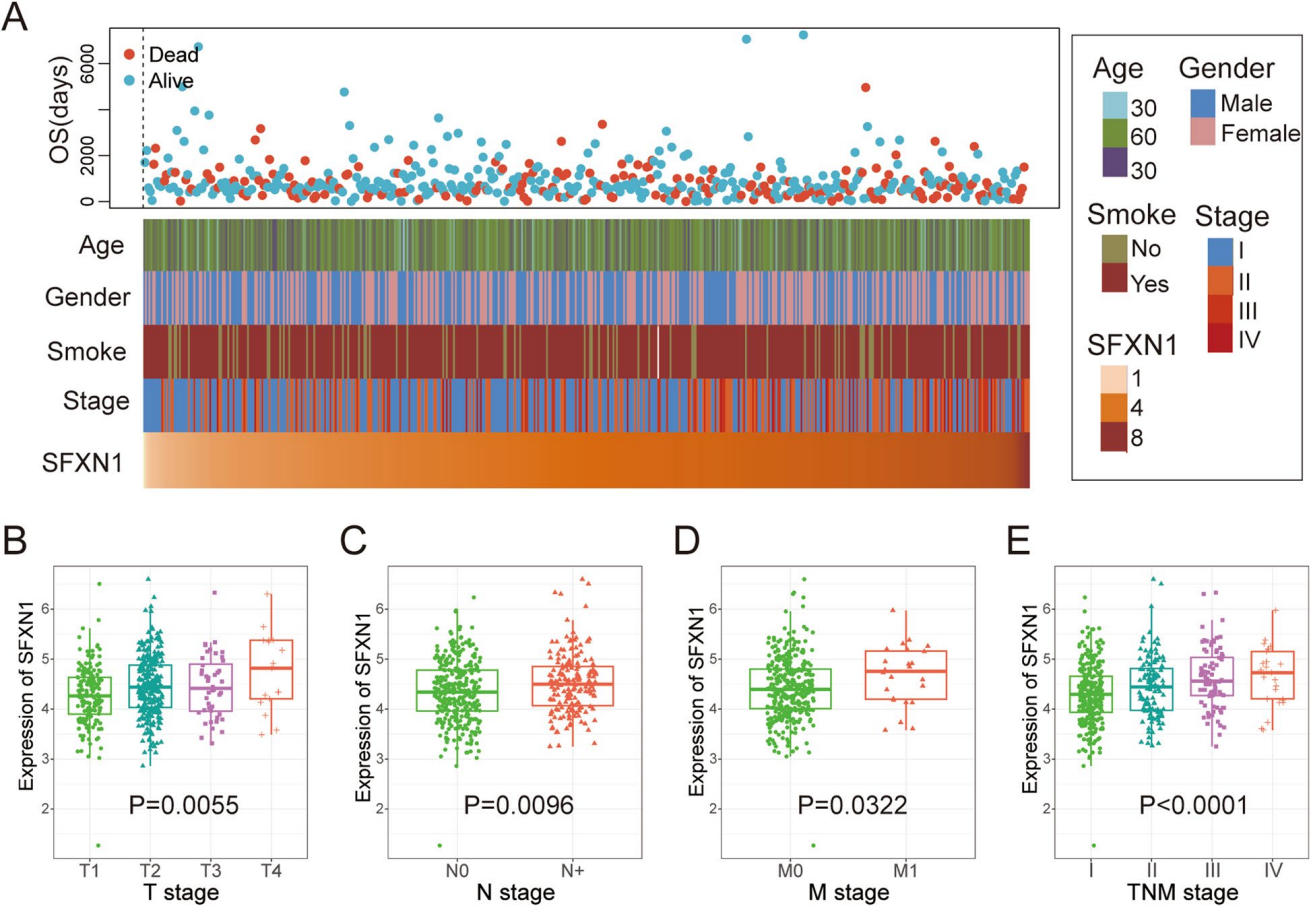
Associations of SFXN1 expression with clinicopathological characteristics in LUAD patients. **A** Distribution of SFXN1 expression in patients with age, gender, smoking history, clinical stage and patient status. **B-E** SFXN1 differential expression in T-stage, N-stage, M-stage and TNM-stage

In this research, we investigated SFXN1 gene expression profiles in 492 LUAD patients from the TCGA database and analyzed their correlation with age, gender, smoking history, and TNM stage (Fig. 2A). Our data revealed that high SFXN1 expression was strongly associated with larger tumor sizes (*P* = 0.0055), lymph node involvement (*P* = 0.0096), distant organ metastasis (*P* = 0.0322), and advanced TNM staging (*P* < 0.0001) among these patients (Fig. 2B-E).

### Differentially expressed genes of SFXN1 by functional enrichment analysis

**Fig. 3:**
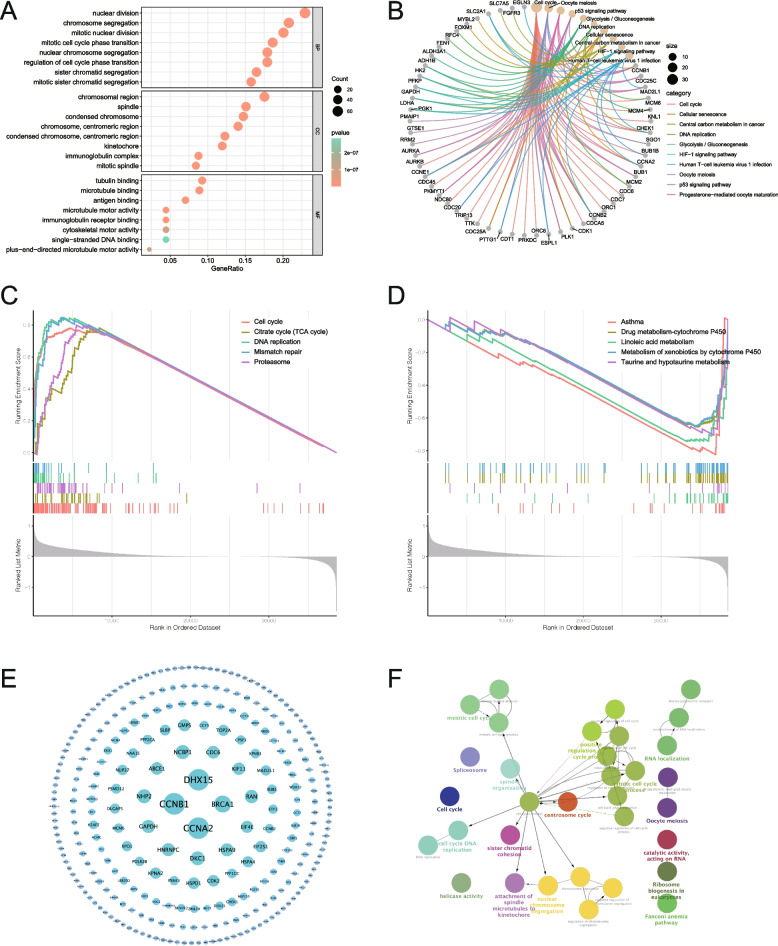
Functional enrichment analysis of SFXN1 in LUAD. **A** Top 8 terms of SFXN1 co-expressed genes based on the GO analysis, including biological process (BP), cellular component (CC), and molecular function (MF) terms. **B** KEGG enrichment pathways of SFXN1 associated genes. **C-D** Top 5 pathways of SFXN1 upregulated or downregulated related genes. **E** PPI network of SFXN1 co-expressed genes. **F** Enriched signaling pathways of SFXN1 related genes and the interrelationship among pathways

To establish the potential functions of SFXN1 in LUAD, we conducted a comprehensive analysis of signaling pathways and biological functions of SFXN1 co-expressed genes in LUAD. Our GO analysis discovered that SFXN1 high expression related genes were prominently enriched in cell cycle-related biological processes (BP), such as mitosis and chromosome segregation. Additionally, molecular functions (MF) were enriched in antigen binding, immunoglobulin binding, and microtubule binding activities (Fig. 3A). KEGG pathway enrichment analysis revealed that SFXN1-associated genes were significantly enriched in numerous cancer-related pathways, such as central carbon metabolism, cell cycle regulation, cellular senescence, DNA repair, HIF-1α signaling, p53 signaling, and glycolysis/ gluconeogenesis (Fig. 3B, all *P* < 0.05). Among them, the top five pathways linked with SFXN1 upregulated genes enriched the cell cycle, TCA cycle, DNA repair, mismatch repair, and proteasome pathways (Fig. 3C), while downregulated genes were mainly enriched in the pathways outlined in Fig. 3D. Subsequently, the PPI network revealed CCNB1, CCNA2, BRCA1, and DHX15 as the top four proteins that significantly interacted with SFXN1 (Fig. 3E). Previous research has demonstrated that CCNB1 plays a crucial role in mediating cell cycle progression by reprogramming energy metabolism in tumor adaptive resistance [26]. Furthermore, CCNB1 and CCNA2 were vital in regulating cell cycle progression, while BRCA1 was involved in DNA repair, damage, and chromatin remodeling [27] and DHX15 overexpression has been shown to promote proliferation and tumor metastasis, particularly in lung cancers [28]. Figure 3 F illustrated the interrelationships between the enriched signaling pathways associated with SFXN1-related genes.

**Fig. 4:**
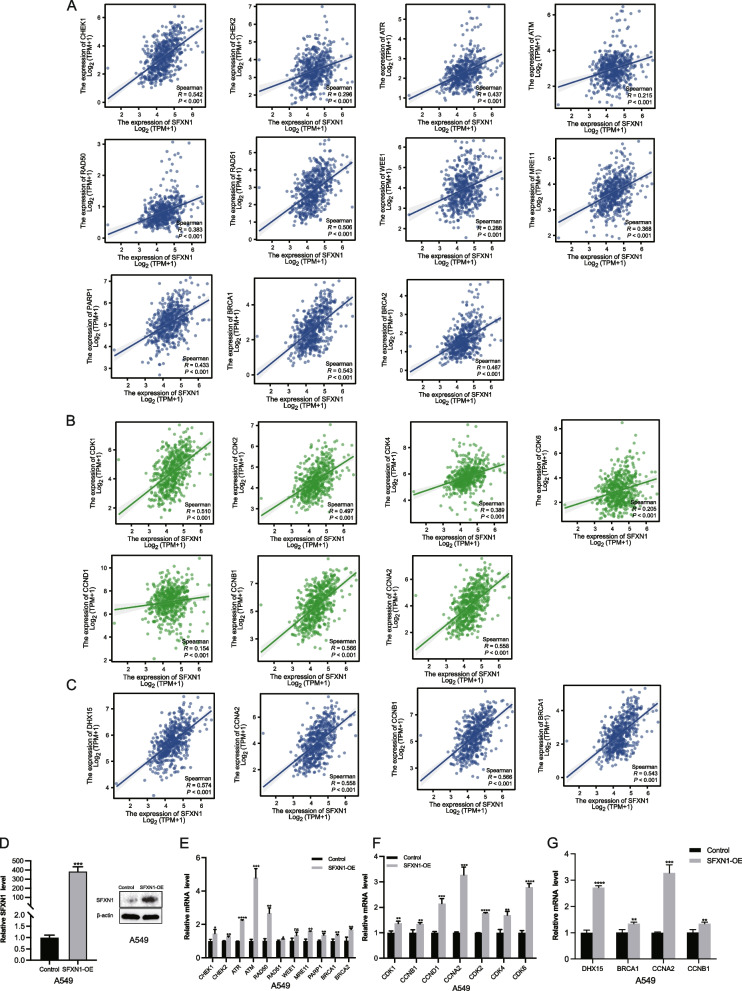
Association of SFXN1 with genes related to cell cycle and DNA repair pathways in vitro. **A-C** Correlation between SFXN1 and genes expression in TCGA database. **D** The efficiency of SFXN1 overexpression in A549 cell line. **E**, **F** qRT-PCR analyses of cell cycle and DNA repair related genes expression in A549 cell infected with control or SFXN1-overexpression lentivirus. **G** Validation of PPI results by qPR-PCR in A549 cell. Note: the blot was cropped at appropriate molecular size markings place in Fig. 4D

We analyzed the correlation between SFXN1 and these genes in TCGA database. The results showed that SFXN1 was related to cell cycle genes and DNA damage repair genes (Fig. 4A-C). Therefore, we detected key genes of the above pathways in A549 cell. First, we evaluated the efficiency of SFXN1 overexpression by RT-qPCR and western blot. The SFXN1 expression at mRNA and protein levels was higher by overexpression vector (Fig. 4D). qRT-PCR results showed that the mRNA levels of DNA damage repair genes CHEK1, CHEK2, ATR, ATM, RAD50, RAD51 and PARP1 were increased in SFXN1 overexpression group (Fig. 4E). Then, CDK1, CDK4, CDK6, CCND1 and CCNB1 in SFXN1 overexpression group were significantly higher than those in control group (Fig. 4F). Also, DHX15 was upregulated after SFXN1 overexpression in A549 cell (Fig. 4G). The results indicated that SFXN1 might interact with these genes to promote the development of LUAD.

### Mutational expression profile of SFXN1 in LUAD

**Fig. 5:**
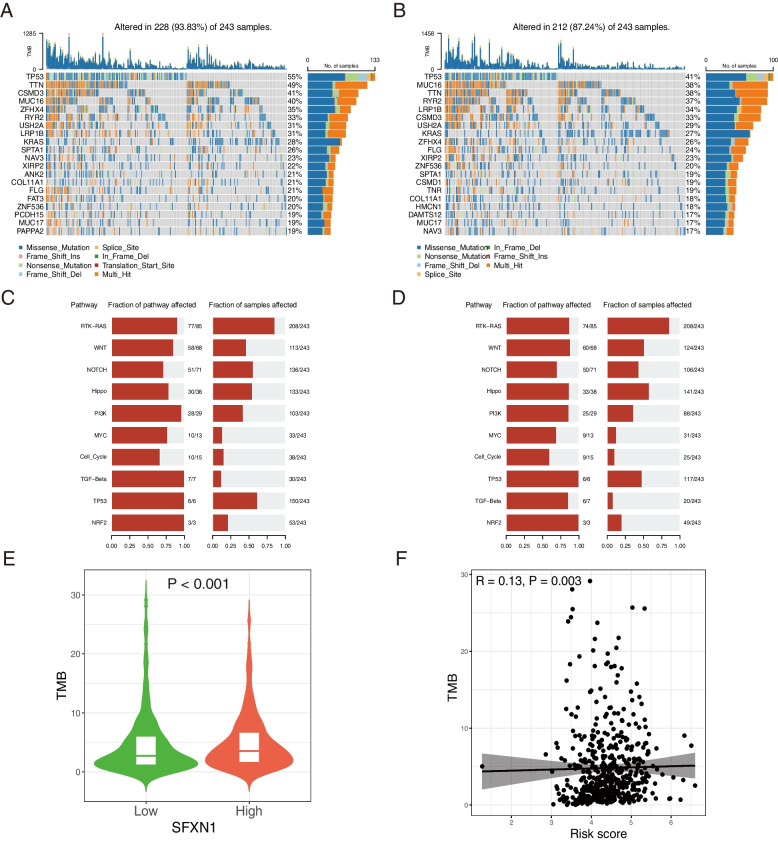
Genetic variations of SFXN1 in LUAD from TCGA database. **A-B** Mutation frequencies of high (**A**) and low (**B**) expression group of SFXN1 in 243 LUAD patients according to the TCGA dataset. Each column represents one patient, the bar on the top represents the TMB, and the numbers on the right mean the mutation frequency of the top 20 common genes in the corresponding group. **C-D** Enriched pathways of SFXN1 mutation in high (**C**) and low (**D**) expression groups. **E** TMB difference between SFXN1 low and high group. **F** Correlation of TMB with the prognostic risk score in LUAD patients

Somatic SFXN1 gene mutations were detected in 243 patients with LUAD from the TCGA database. Waterfall plot revealed 93.83% (228/243) somatic mutation rate in patients with high expression, TP53 and TTN exhibited higher mutation frequency. The low expression group had a somatic mutation rate of 87.24% (212/243), with MUC16 and TTN exhibiting higher mutation frequency apart from TP53 (Fig. 5A, B). Various SFXN1 expression groups showed significant enrichment of pathways, with SFXN1 exhibiting somatic gene mutations in cancer-related signaling pathways like RTK-RAS, WNT, NOTCH, Hippo and PI3K which indicates its critical role in cancer development (Fig. 5C, D). Notably, patients with high SFXN1 expression exhibited a significantly higher TMB than those with low expression (*P* < 0.001). Furthermore, the TMB displayed a positive correlation with the prognostic risk score of LUAD patients (Fig. 5E, F), indicating poorer prognosis in the SFXN1 overexpression group.

### SFXN1 involved in regulating the immune microenvironment of LUAD

**Fig. 6:**
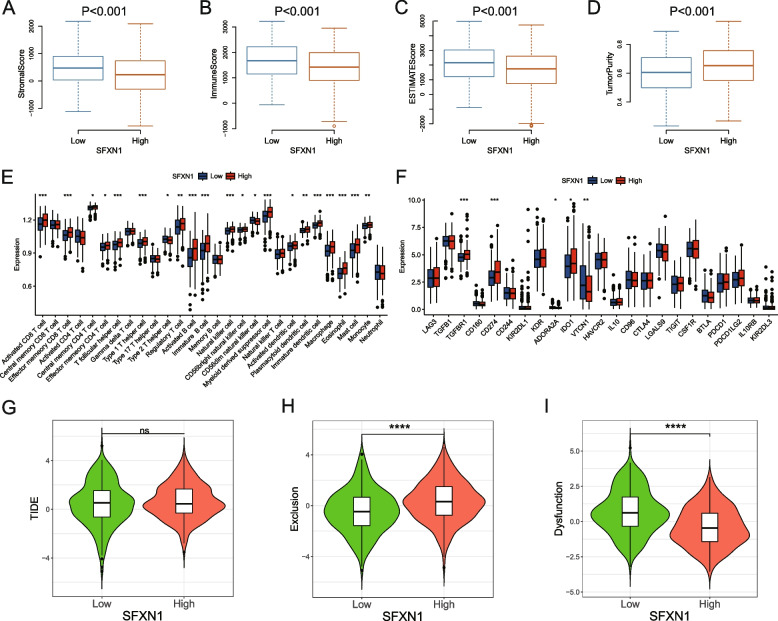
Correlation of SFXN1 expression with immune cell infiltration, inhibitory immune molecules, and immunotherapy response in the TCGA LUAD dataset. **A-B** The difference of stroma score (**A**), immune score (**B**), estimate score (**C**) and tumor purity (**D**) between low and high SFXN1 expression group. **E-F** The 28 immune cell composition (**E**) and immune exhaustion molecules (**F**) expression in different SFXN1 expression in LUAD patients. **G-I** Immunotherapy response biomarkers, including tumor immune dysfunction and exclusion (TIDE) score (**G**), T-cell exclusion score (**H**) and T-cell dysfunction score (**I**), between SFXN1 low and high expression group

Patients with SFXN1 high expression have relatively lower stromal score, immune score, and estimate score compared with the low expression group, which was consistent with the observation that patients with high SFXN1 expression had higher tumor purity (*P* < 0.001, Fig. 6A-D) and SFXN1 upregulation may increase malignancy in patients. Then, we used the CIBERSORT algorithm to analyze immune cell infiltration in different SFXN1 expression groups from the TCGA database. The results manifested that activated CD8 + T cells, activated B cells, natural killer cells (NKs), activated dendritic cells (DCs), macrophages, myeloid-derived suppressor cells (MDSC_S_) and mast cells (MCs) were considerably upregulated in the high SFXN1 expression group (Fig. 6E). We also explored the relationship between differential SFXN1 expression and inhibitory immune molecules in LUAD and observed significant upregulation of CD274, IDO1, and TGFBR1 in the high SFXN1 expression group (Fig. 6F). Furthermore, we analyzed the correlation between SFXN1 expression and the efficacy of immunotherapy and found no significant difference in TIDE score between the differential SFXN1 expression groups (Fig. 6G). However, T-cell dysfunction score was lower in the high SFXN1 expression group (Fig. 6I). Interestingly, patients with high SFXN1 expression had a significantly higher T-cell exclusion rate (Fig. 6H).

To comprehensively investigate the role of SFXN1 in the LUAD immune microenvironment, we analyzed the correlation between SFXN1 expression and immune microenvironment factors through TISIDB. Supplementary Fig. 1 presents the molecules with the strongest correlation with SFXN1: Lymphocyte: activated CD4 + T cell (*R* = 0.246, *P* = 4.68e-18), Eosinophil (*R*=-0.338, *P* = 3.893e-15) (Fig. S1A); Immunostimulator: PVR (CD155) (*R* = 0.246, *P* = 1.69e-08), TNFSF13 (*R*=-0.367, *P* < 2.2e-16) (Fig. S1B); Immunoinhibitor:CD274 (*R* = 0.196, *P* = 7.61e-06), VTCN1(*R*=-0.266, *P* = 8.82e-10) (Fig. S1C); MHC molecular: TAP1(R = 0.205, *P* = 2.85e-06), HLA-DBP1(*R*=-0.274, *P* = 2.83e-10) (Fig. S1D); Chemokine: CCL26(*R* = 0.236, *P* = 6e-08), CCL14 (*R*=-0.398, *P* < 2.2e-16) (Fig. S1E); Chemokine receptor:CX3CR1 (*R*=-0.32, *P* = 1.2e-13), CCR7 (*R*=-0.222, *P* = 3.45e-07) (Fig. S1F). These results indicated that SFXN1 may play a complex regulatory role in the immune microenvironment of LUAD.

### SFXN1 overexpression is associated with efficacy of various antitumor Drugs

**Fig. 7:**
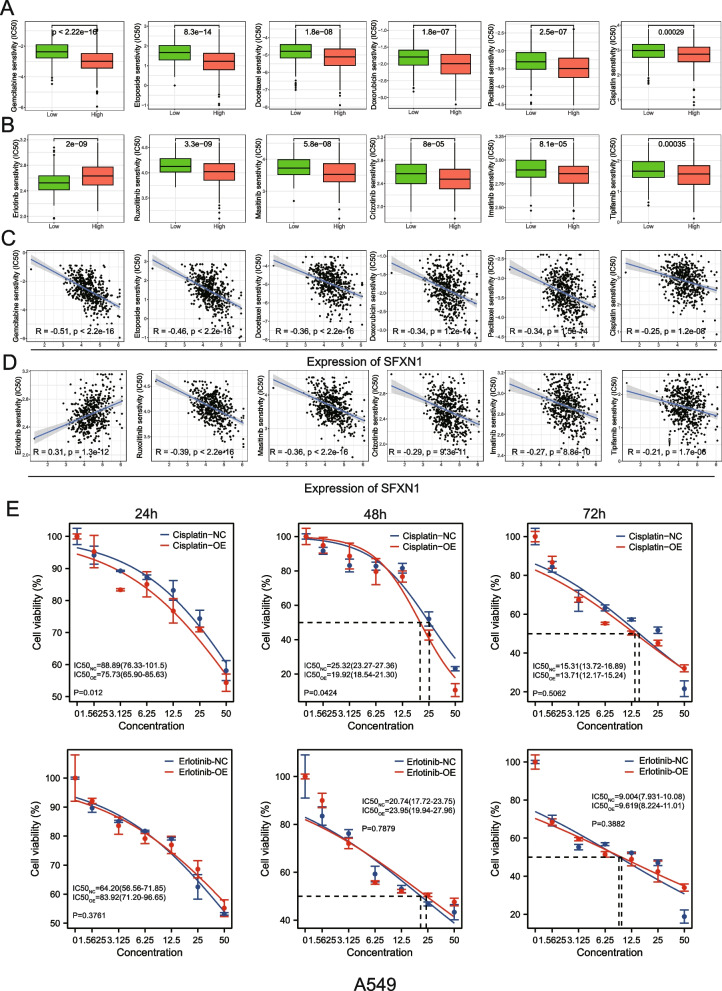
Evaluation of drug sensitivity of different SFXN1 expression groups in LUAD. **A-B** Comparison IC50 values between low and high SFXN1 expression groups based on the TCGA LUAD dataset. **C-D** Correlation of SFXN1 expression with estimated IC50 values of chemotherapy drugs (**C**) and targeted drugs (**D**). **E** CCK-8 analyses of the cell viability of erlotinib and cisplatin in A549 cell infected with control or SFXN1-overexpression lentivirus at 24h, 48h and 72h

To investigate the clinical relevance of SFXN1 expression, we examined the correlation of its expression with the IC50 values of chemotherapy and targeted drugs in the TCGA database (screening criteria: *P* < 0.05, |R| > 0.20). The results proved that upregulated SFXN1 expression was significantly associated with lower IC50 values for almost all clinical chemotherapy drugs and targeted therapies, except for erlotinib, indicating that patients with upregulated SFXN1 expression are presumably more receptive to anti-tumor drugs (Fig. 7A, B). Further analysis revealed a significant negative correlation between SFXN1 expression levels and IC50 values for almost all drugs, except for erlotinib (Fig. 7C, D). To verify the above results, we tested the sensitivity of erlotinib and cisplatin in A549 cells overexpressing SFXN1 by CCK8 assay. The results showed that the inhibition of the drug on the cells was time-dependent and dose-dependent. In addition, compared with control cells, SFXN1 overexpressed cells were relatively sensitive to cisplatin, while Erlotinib had a higher IC50 in SFXN1 overexpression cell than the control group (Fig. 7E), which was consistent with the results of drug sensitivity analysis.

#### Visualization SFXN1 expression in immune cells of LUAD

**Fig. 8:**
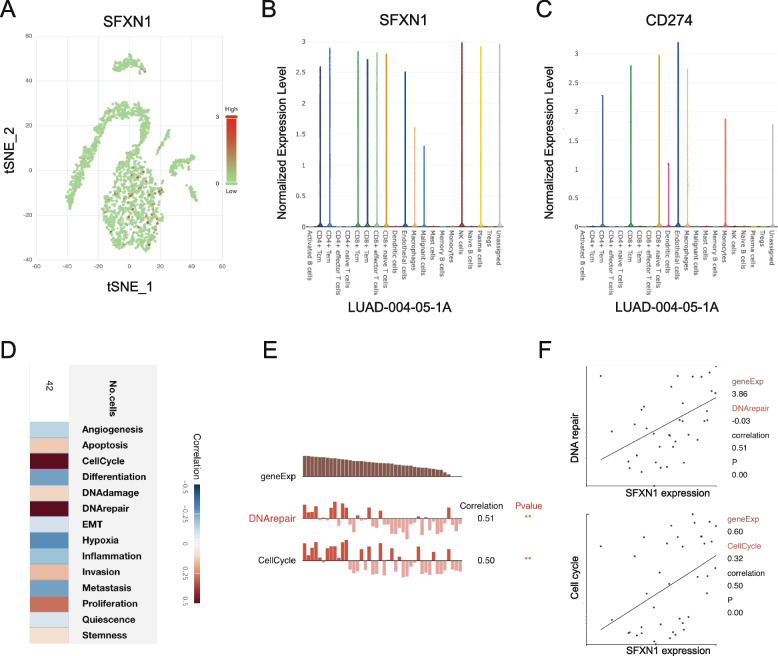
Visualization SFXN1 expression on immune cells in LUAD patients. **A** t-SNE plot of different SFXN1 expression on immune cells from LUAD-004-05-1A patient. **B-C** SFXN1 and CD274 expression on different immune cell types in LUAD-004-05-1A. **D** Enriched signaling pathways of different SFXN1 expression on immune cells. **E-F** Correlation of cell cycle and DNA repair with SFXN1 expression

We examined the expression of SFXN1 in the tumor microenvironment by analyzing single-cell sequencing data from an online database of LUAD patients (Fig. 8A). We then investigated the expression of CD274 and SFXN1 across various immune cell populations. The results showed that SFXN1 was predominantly expressed in CD4 + effector memory T cells (CD4 + Tem), CD4 + central memory T cells (CD4 + Tcm), CD8 + central memory T cells (CD8 + Tcm), CD8 + naive T cells, endothelial cells, macrophages, and natural killer (NK) cells, which was consistent with the distribution pattern of CD274 expression (Fig. 8B, C), suggesting that SFXN1 may be implicated in tumor immune evasion mechanisms. Additionally, we observed that SFXN1 expression was positively associated with several cellular pathways involved in cell cycle, DNA repair, proliferation, invasion, DNA damage, and stemness, which were consistent with our previous analysis using KEGG enrichment (Fig. 8D). Notably, SFXN1 expression showed significant positive correlations with DNA repair (*R* = 0.51, *P* < 0.01) and cell cycle (*R* = 0.50, *P* < 0.01) signal enrichment scores (Fig. 8E, F).

### SFXN1 expression independently predicts survival prognosis of LUAD patients

**Fig. 9:**
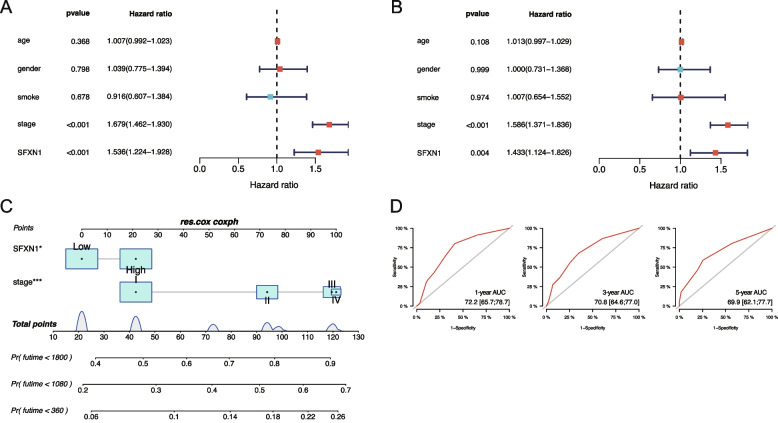
Prognostic significance of SFXN1 in the TCGA LUAD dataset. **A-B** Univariate and multivariate Cox regression analyses of clinical parameters for OS in the TCGA LUAD cohort. **C** The prognostic nomogram based on SFXN1 expression and TNM stage. **D** The ROC curves of 1-year, 3-year, 5-year OS in the TCGA dataset

To analyze the prognostic significance of SFXN1 expression in LUAD patients, we conducted Kaplan-Meier survival and Cox regression analyses based on the TCGA database. Our findings revealed that increased SFXN1 expression was associated with significantly lower overall survival (OS) among patients. Additionally, multivariate Cox regression analysis demonstrated that SFXN1 expression and TNM stage were independent predictors of patient survival regardless of age, gender and smoking history (Fig. 9A, B). We also constructed a predictive nomogram model that integrated SFXN1 expression and TNM stage to forecast patients’ 1-year, 3-year, and 5-year OS (Fig. 9C). ROC curve analyses further indicated that the predictive accuracy of our model was superior to that of TNM stage alone (Fig. 9D), with the area under curve (AUC) values for ROC based on SFXN1 expression and TNM stage at 1-year, 3-year, and 5-year being 77.2, 70.8, and 69.9, respectively.

#### Expression and clinical value of SFXN1 in validation set

**Fig. 10:**
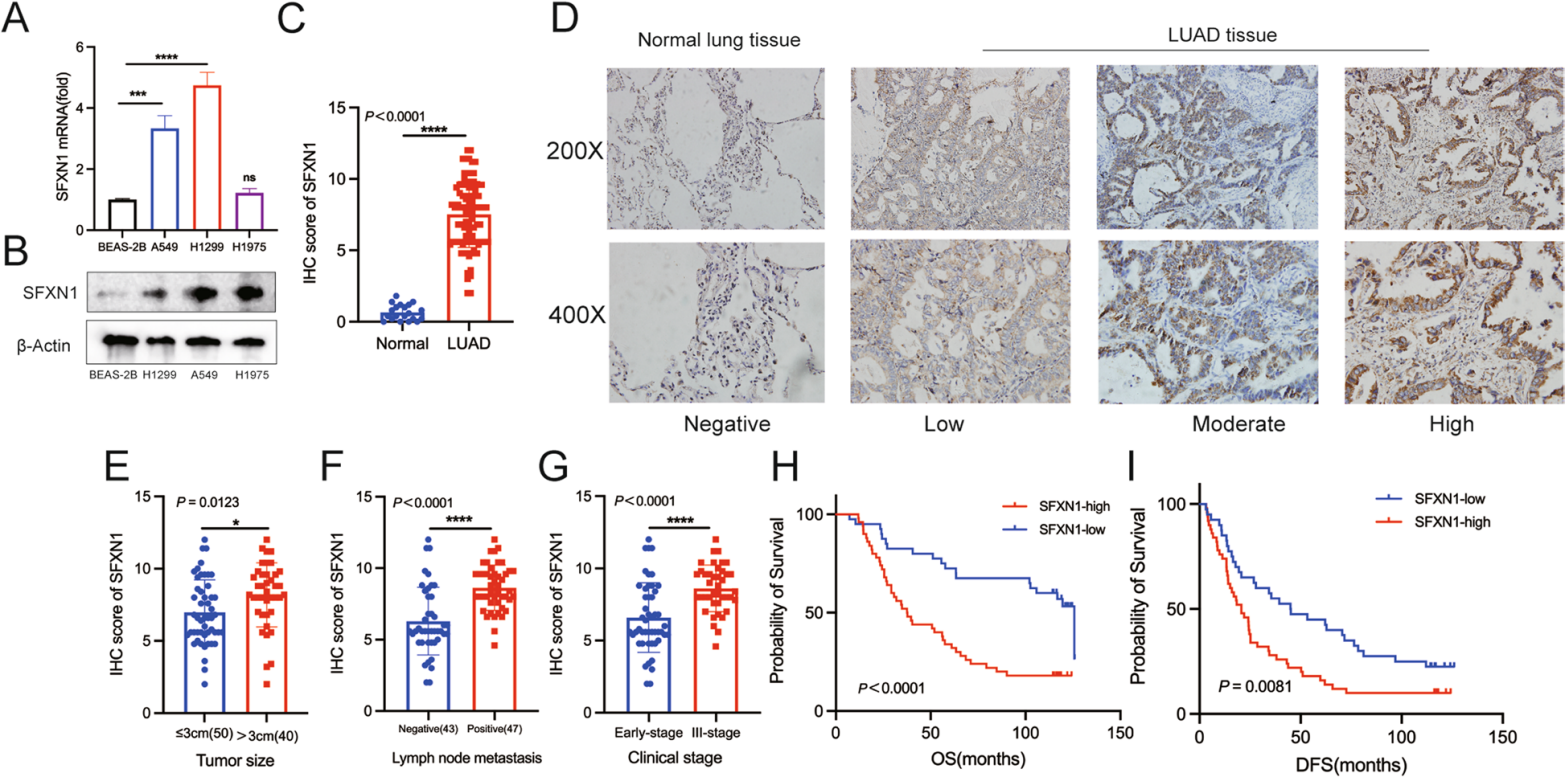
Prognostic value of SFXN1 protein in validation set. **A-B** The mRNA and protein level of SFXN1 in different LUAD and normal lung cell lines. **C** The differential expression of SFXN1 in normal tissues (*n* = 20) and LUAD tissues (*n* = 90). **D** The typical IHC staining images of SFXN1 in normal lung tissues and LUAD tissues. **E-G** Association of SFXN1 expression with clinical features, including tumor size (**E**), lymph node metastasis status (**F**) and clinical stage (**G**). **H-I** The KM curves about the correlation between SFXN1 expression and overall survival (**H**) and disease-free survival (**I**). (*P < 0.05, **P < 0.01 and ****P < 0.0001, ns: no significant). Note: the blot was cropped at appropriate molecular size markings place in Fig. 10B

We conducted experiments using qRT-PCR and WB to validate the results obtained from the TCGA database, using both normal and LUAD cells. Our findings indicated a statistically significant increase in SFXN1 expression in LUAD cells compared to BEAS-2B (*P* = 0.0001 for A549 vs. BEAS-2B; *P* < 0.0001 for H1299 vs. BEAS-2B), but not in H1975 (*P* = 0.79) (Fig. 10A). WB results showed that SFXN1 was upregulated in all LUAD cells at the protein level (Fig. 10B). Moreover, IHC results also verified a considerably higher SFXN1 expression in LUAD samples compared to adjacent normal lung tissues (*P* < 0.0001) (Fig. 10C, D). The baseline information of our clinical data showed in Table 2. Furthermore, we analyzed the association between SFXN1 expression and clinical characteristics of LUAD patients. The results showed that SFXN1 expression was significantly higher in patients with locally advanced stage (*P* < 0.0001), lymph node metastasis (*P* < 0.0001), and larger tumor size (*P* = 0.0123) than in patients with early-stage disease, no lymph node metastasis, and T1 stage disease (Fig. 10E-G). Our analysis of clinical data produced results identical to the TCGA cohort, showing that LUAD patients with high SFXN1 expression (*n* = 40) had significantly shorter OS and disease-free survival (DFS) than those with low expression (*n* = 50) (Fig. 10H, I). Univariate and multivariate analysis revealed that SFXN1 was an independent prognostic factor for OS in LUAD patients, but not DFS (Table 3 and Supplementary Table 2).

**Fig. 11:**
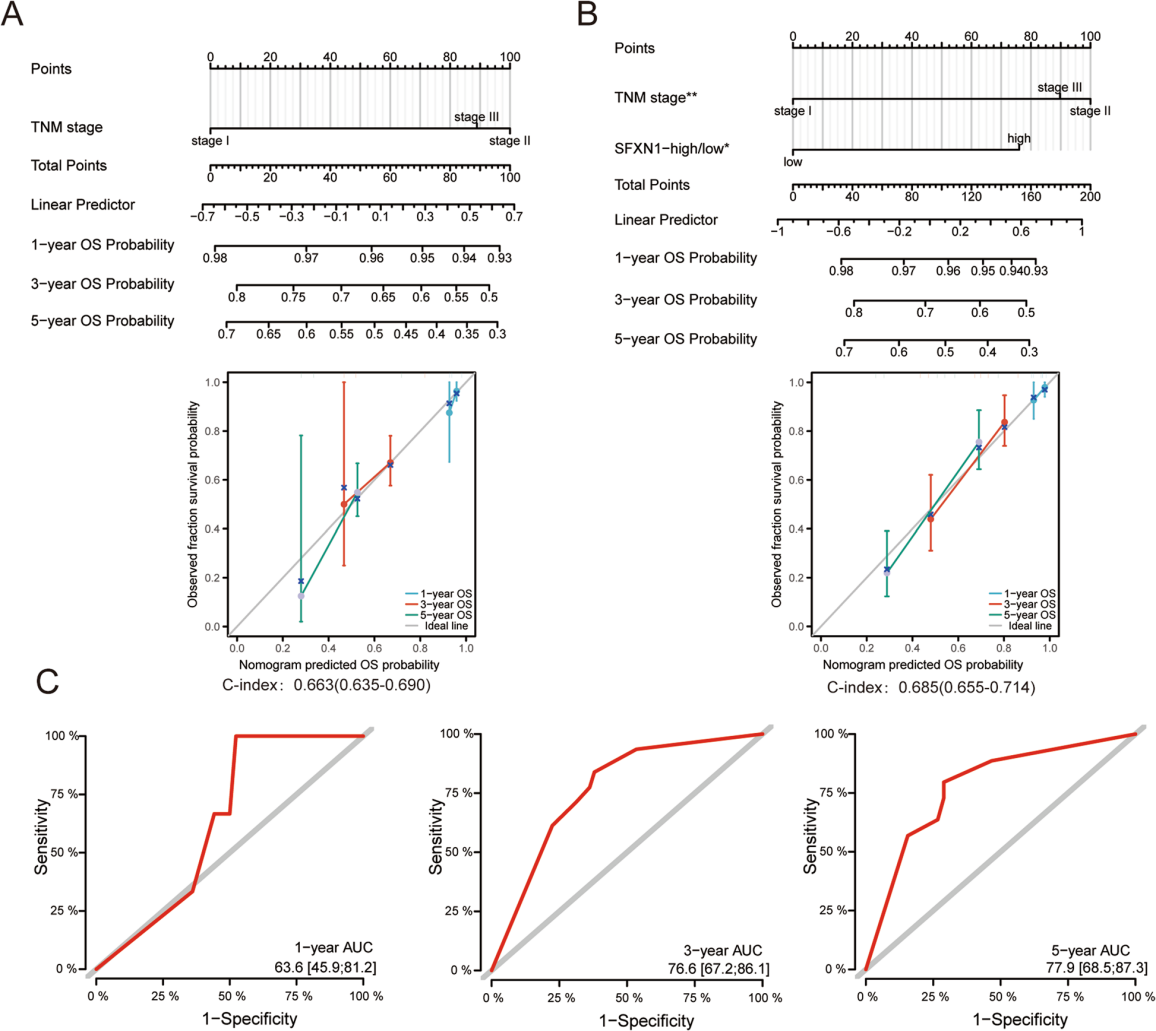
Nomogram based on SFXN1 IHC score and TNM stage in validation set. **A-C** The nomogram, calibration curves and ROC curves for OS

Finally, a nomogram comprised of SFXN1 expression and TNM staging, underscored the benefits of combining these factors to better predict the survival of LUAD patients (OS:C-index = 0.685; DFS:C-index = 0.674) (Fig. 11A, B, Fig. S2). ROC curve analyses showed that the AUC values based on SFXN1 IHC score and clinical stage at 1-year, 3-year, and 5-year OS being 63.6, 76.6, and 77.9, respectively (Fig. 11C).

**Table 2:** The clinical characteristics in validation set

Clinical characteristics	Number (90)	%
**Age(year)**		
Median (Min, Max)	59 (31–79)	-
**Gender**		
Male	47	52.22
Female	43	47.78
**ECOG-PS**		
0	35	38.89
1	55	61.11
**Smoking history**		
Non-smoker	50	55.56
Smoker	40	44.44
**Clinical stage**		
Stage I	41	45.56
Stage II	8	8.89
Stage III	41	45.56
**Tumor size**		
T1	50	55.56
T2	36	40.00
T3 + T4	4	4.44
**Lymph node metastasis**		
N0	43	47.78
N1	7	7.78
N2	40	44.44

**Table 3:** The Cox regression analysis among clinical traits and OS in validation set

Characteristics	N	Univariate analysis		Multivariate analysis	
		Hazard ratio (95% CI)	*P* value	Hazard ratio (95% CI)	*P* value
**Gender**			0.489		
Male	47	Reference			
Female	43	0.835 (0.500–1.394)	0.490		
**Age**	90	1.006 (0.978–1.036)	0.663		
**Smoking history**			0.527		
Non-smoker	50	Reference			
Smoker	40	1.181 (0.707–1.972)	0.526		
**ECOG-PS**			0.134		
0	35	Reference			
1	55	1.499 (0.874–2.572)	0.142		
**TNM stage**			**< 0.001**		
I	41	Reference		Reference	
II	8	3.840 (1.590–9.275)	**0.003**	3.265 (0.656–16.256)	0.149
III	41	3.308 (1.867–5.864)	**< 0.001**	2.618 (0.634–10.801)	0.183
**T stage**			**0.017**		
<3 cm	50	Reference		Reference	
≥ 3 cm	40	1.877 (1.121–3.143)	**0.017**	1.149 (0.646–2.043)	0.637
** N stage**			**< 0.001**		
Negative	43	Reference		Reference	
Positive	47	3.122 (1.814–5.374)	**< 0.001**	0.888 (0.227–3.474)	0.864
**IHC score of SFXN1**	90	1.253 (1.121–1.402)	**< 0.001**	1.178 (1.032–1.345)	**0.016**

## Discussion

Given the expanding global population and increasing life expectancy, the alarming incidence cancer in 2030 was predicted to reach 20.3 million worldwide, and is poised to become the main cause of morbidity and mortality across all regions of the world in the next decade [29]. Specifically, the highest lifetime risk of developing of cancers is lung cancer, the most common type of cancer death in 94 countries, in both high and low Human Development Index(HDI) regions [29]. What’s worse, despite many years of efforts, the metabolic reprogramming of cancer cells is far beyond fully understanding and there has been limited clinical progress in addressing this mechanism [30] due to the complex features of aberrant cancer metabolism.

Recent studies have reported a significant upregulation of SFXN1 mRNA level in various malignancies compared with normal tissues [15]. Despite some studies investigating the differential expression and function of the SFXN family genes in cancer, no evidence regarding the association of SFXN1 expression with immunotherapy response has been identified. Therefore, this study employed data mining techniques alongside clinical validation to identify promising prognostic biomarkers within the SFXN family for LUAD. By conducting a comprehensive and comparative analysis of the compound genes, we aim to unveil their interrelations and pinpoint novel biomarkers. Targeting a specific gene in the SFXN family aligns with “Precision Medicine” and has the potential to induce maximally synergistic effects in clinical practice. Our results showed a significant increase in the expression of SFXN1 and SFXN4 in LUAD. However, only SFXN1 had a significant impact on the overall survival of LUAD patients. Previous research has shown that all SFXN family members are present in pancreatic islet cells while SFXN3, in particular, is a crucial carrier molecule for the differentiation and regeneration of pancreatic β-cells [31]. Likewise, while many SFXN family members are highly expressed in LUAD, SFXN1 exerts a significant influence on the development and progression of the disease. Our investigation was focused on SFXN1 as a potential prognostic biomarker for LUAD. We found that SFXN1 expression was correlated with clinical stage, tumor size, lymph node invasion, and distant metastasis. Our study also demonstrated that patients with high SFXN1 expression had a significantly higher TMB, which is consistent with previous findings linking high TMB to a poor prognosis for non-small cell lung cancer [32]. Furthermore, both univariate and multivariate Cox regression analyses confirmed that SFXN1 remained a prognostic indicator associated with overall OS, indicating that this gene acts as an independent predictor for LUAD. After extending the follow-up period, the accuracy of nomogram based on the SFXN1 protein expression and clinical stage has significantly improved, indicating a more reliable prediction of the outcome. This extension allowed for a more thorough analysis of the data and additional insights into the long-term effects of the intervention.

The KEGG pathway and GO analysis of SFXN1 interactive genes in this study evidenced that SFXN1 participates in the cell cycle, including DNA replication, meiosis, and signaling pathways such as p53 and HIF-1 pathways. As mitochondria carry their own DNA (mtDNA) in eukaryotic cells, they also regulate the cell cycle and mitosis through their metabolic activities [10]. Mutations in mtDNA can generate a more favorable metabolic profile in rapidly proliferating tumor cells, accelerating cancer progression and metastasis [33]. Thus, SFXN1 may exacerbate LUAD progression by promoting cancer cell proliferation, inhibiting apoptosis, and activating invasion and metastasis pathways. Therefore, altered expression levels of SFXN1 in LUAD might be a critical factor, and further investigation is necessary to determine whether it acts as a driver or passenger oncogene. Functionally, the GO molecular function (MF) analysis revealed that SFXN1 has a broad function in cytoskeletal movement and microtubule binding, both of which require mitochondrial energy in process.

Mitochondria serve as the primary energy-producing organelles in cells and have key roles in generating reactive oxygen and regulating iron metabolism. Mutation of the SFXN1 gene, which is responsible for facilitating the transportation of iron into the mitochondria, can result in pathologic accumulation of iron within erythrocytes, as observed in flexed-tail (f/f) mice [34]. In addition, researchers discovered that SFXN1 is a mitochondrial iron transporting protein that carries excess free iron into the mitochondria, resulting in mitochondrial damage and subsequent cardiomyocyte hypertrophy in the long term [35]. Sousa and colleagues also demonstrated that erythrocytes in patients with iron overload exhibit elevated ATPase activity [36]. These findings highlight the critical role of proper regulation of iron metabolism in mitochondria for maintaining cellular function. Therefore, SFXN1 might facilitate iron transport by boosting the ATPase activity, which needs additional experimental verification. Additionally, during the last decade, metabolic reprogramming has been the most significant factor observed among the pathways of central carbon metabolism in cancer cells [37]. Our study’s KEGG enrichment analysis revealed that SFXN1 participated in the folate-dependent central carbon metabolism in cancer, which is consistent with the remarkable work that identified SFXN1 as the primary SFXN carrier protein for transporting serine into mitochondria during the one-carbon metabolism process [15]. Our study also found that tumor purity was higher in LUAD patients with SFXN1 overexpression, indicating that there were fewer infiltrating immune cells in the tumor microenvironment. This may be one of the reasons for the poorer prognosis of patients with SFXN1 upregulation. A growing body of evidence has shown that mitochondria not only serve as the “energy factories” of immune cells, but also modulate immune responses by regulating metabolic and physiological states in different types of immune cells [38, 39]. Serine is a primary source of one-carbon units and abnormal metabolism of serine is closely related to cancer progression [40]. Studies have confirmed that restricting serine can inhibit tumor growth in mice [41]. Thus, inhibiting the expression of SFXN1, which is the key transporter of serine into mitochondria [15], may reduce tumor development. Importantly, our study found that a large number of MCs, eosinophils, macrophages, MDSCs, and Treg cells infiltrated the SFXN1 high-expression group, all of which can shape an immunosuppressive microenvironment and promote tumor growth under certain conditions. For example, MCs release FGF-2, NGF, PDGF, VEGF, IL-8, and IL-10, which promote the expansion of tumor cells [42]. Li et al. demonstrated that eosinophils promote tumor cell migration and bone metastasis by secreting C-C motif chemokine ligand 6 (CCL6) in mice [43]. MDSCs have been shown to suppress immune responses and protect tumor cells from attack, making them valuable prognostic biomarkers and potential targets for anti-cancer therapies [44]. Macrophages are classified into pro-inflammatory (M1) and immunosuppressive (M2) macrophages, with M2-polarized macrophages associated with poorer prognosis in cancer patients. Therefore, SFXN1 may modulate the tumor immune microenvironment by directly or indirectly influencing the infiltration of immune cells. Importantly, we found that SFXN1 overexpression was associated with higher expression of immune checkpoints CD274 (PD-L1) and IDO1. CD274 is predominantly expressed by tumor cells, binding with programmed cell death-1(PD-1) on the surface of T cells and triggering immune escape [44]. IDO1, indoleamine 2, 3-dioxygenase 1, is a widely expressed enzyme in human cancers that metabolizes tryptophan to kynurenine, which mainly interacts with effector T cells to impair their antitumor effects and facilitate immune escape [45, 46]. Additional studies indicated that IDO1 enhances the proliferation of regulatory T cells (Tregs) and activates MDSCs [47, 48]. Therefore, these findings suggest that the high expression of SFXN1 in the tumor immune microenvironment may lead to immunosuppression and possibly compromise the efficacy of immunotherapeutic strategies.

While our research has confirmed the role of SFXN1 in LUAD using publicly available cohorts and our own clinical samples, further investigations are required to fully understand the biological processes and oncogenic mechanisms that involve SFXN1, both in vitro and in vivo.

## Conclusion

This study identified a significant difference in the expression levels of SFXN family genes between LUAD and normal samples. Moreover, an analysis of the association between SFXN1 and clinicopathologic characteristics, overall survival status, drug sensitivity, and immune cell infiltration demonstrated that SFXN1 plays a pivotal role in both cancer development and regulation of the immune microenvironment. In summary, SFXN1 has the potential to act as a valuable prognostic biomarker and new therapeutic target for LUAD.

### Supplementary Information

** Additional file 1:** Supplementary figures, tables and WB original blots. **Fig. S1. **Association of SFXN1 expression with lymphocytes, immunomodulators, MHC molecules, chemokines and chemokine receptors. **Fig. S2.** Nomogram based on SFXN1 IHC score and TNM stage for DFS in validation set. **Supplementary Table 1. **Other primes used in this study are as follows. **Supplementary Table 2.** The Cox regression analysis among clinical traits and DFS in validation set. WB original blots in supplementary information.

## Data Availability

The datasets presented or analysed during this study are freely available in the TCGA database (https://portal.gdc.cancer.gov/) and its supplementary information files. The dataset of our clinical data is available from the corresponding author on reasonable request.

## Abbreviations

NSCLC: Non-small cell lung cancer
LUAD: Lung adenocarcinoma
SFXN1: Sideroflexin1
TCGA: The Cancer Genome Atlas
qRT-PCR: Real-time quantitative PCR
WB: Western Blotting
IHC: Immunohistochemistry
NKs: Natural Killer cells
DCs: Dendritic cells
GEO: Gene Expression Omnibus
CPTAC: Clinical Proteomic Tumor Analysis Consortium
ATCC: American Type Culture Collection
PAGE: Polyacrylamide gel electrophoresis
PVDF: Polyvinylidene fluoride
GO: Gene ontology
GSEA: Gene Set Enrichment Analysis
PPI: Protein-protein interaction
TIDE: Tumor immune dysfunction and exclusion
CGP: Cancer Genome Project
TCR: T-cell receptor
KM: Kaplan–Meier
ROC: Receiver operating characteristic
KIRC: Kidney renal clear cell carcinoma

## References

[1] Siegel RL, Miller KD, Wagle NS, Jemal A (2023). Cancer statistics, 2023. CA Cancer J Clin.

[2] Molina JR, Yang P, Cassivi SD, Schild SE, Adjei AA (2008). Non-small cell Lung cancer: epidemiology, risk factors, treatment, and survivorship. Mayo Clin Proc.

[3] Gridelli C, Rossi A, Maione P (2003). Treatment of non-small-cell lung cancer: state of the art and development of new biologic agents. Oncogene.

[4] Nan X, Xie C, Yu X, Liu J (2017). EGFR TKI as first-line treatment for patients with advanced EGFR mutation-positive non-small-cell Lung cancer. Oncotarget.

[5] Jackson SE, Chester JD (2015). Personalised cancer medicine. Int J Cancer.

[6] Boolell V, Alamgeer M, Watkins DN, Ganju V (2015). The evolution of therapies in non-small cell lung cancer. Cancers (Basel).

[7] Hanahan D, Weinberg RA (2011). Hallmarks of cancer: the next generation. Cell.

[8] Momcilovic M, Jones A, Bailey ST, Waldmann CM, Li R, Lee JT, et al. In vivo imaging of mitochondrial membrane potential in non-small-cell lung cancer. Radiol Imaging Cancer. 2020;2(7782):e204006. 10.1148/rycan.2020204006 PMC7983760 33778706

[9] Weinberg SE, Sena LA, Chandel NS (2015). Mitochondria in the regulation of innate and adaptive immunity. Immunity.

[10] Lennon FE, Salgia R (2014). Mitochondrial dynamics: biology and therapy in lung cancer. Expert Opin Investig Drugs.

[11] Vasan K, Werner M, Chandel NS (2020). Mitochondrial metabolism as a target for cancer therapy. Cell Metab.

[12] Nam HS, Izumchenko E, Dasgupta S, Hoque MO (2017). Mitochondria in chronic obstructive pulmonary disease and lung cancer: where are we now?. Biomark Med.

[13] Zheng H, Ji C, Zou X, Wu M, Jin Z, Yin G (2003). Molecular cloning and characterization of a novel human putative transmembrane protein homologous to mouse sideroflexin associated with sideroblastic anemia. DNA Seq.

[14] Xi D, He Y, Sun Y, Gou X, Yang S, Mao H (2011). Molecular cloning, sequence identification and tissue expression profile of three novel genes Sfxn1, Snai2 and cno from Black-boned sheep (Ovis aries). Mol Biol Rep.

[15] Kory N, Wyant GA, Prakash G, Uit de Bos J, Bottanelli F, Pacold ME, et al. SFXN1 is a mitochondrial serine transporter required for one-carbon metabolism. Science. 2018;362(6416): eaat9528. 10.1126/science.aat9528 PMC6300058 30442778

[16] Chen Q, Wang R, Zhang J, Zhou L (2019). Sideroflexin1 as a novel tumor marker independently predicts survival in lung adenocarcinoma. Transl Cancer Res.

[17] Chen L, Kang Y, Jiang Y, You J, Huang C, Xu X (2022). Overexpression of SFXN1 indicates poor prognosis and promotes tumor progression in lung adenocarcinoma. Pathol Res Pract.

[18] Liu W, Jiang K, Wang J, Mei T, Zhao M, Huang D (2021). Upregulation of gnpnat1 predicts poor prognosis and correlates with immune infiltration in lung adenocarcinoma. Front Mol Biosci.

[19] Wang J, Sun T, Meng Z, Wang L, Li M, Chen J (2021). XPO1 inhibition synergizes with PARP1 inhibition in small cell Lung cancer by targeting nuclear transport of FOXO3a. Cancer Lett.

[20] Chandrashekar DS, Karthikeyan SK, Korla PK, Patel H, Shovon AR, Athar M (2022). UALCAN: an update to the integrated cancer data analysis platform. Neoplasia.

[21] Tang Z, Li C, Kang B, Gao G, Li C, Zhang Z (2017). GEPIA: a web server for cancer and normal gene expression profiling and interactive analyses. Nucleic Acids Res.

[22] Tang Z, Kang B, Li C, Chen T, Zhang Z (2019). GEPIA2: an enhanced web server for large-scale expression profiling and interactive analysis. Nucleic Acids Res.

[23] von Mering C, Huynen M, Jaeggi D, Schmidt S, Bork P, Snel B (2003). STRING: a database of predicted functional associations between proteins. Nucleic Acids Res.

[24] Szklarczyk D, Gable AL, Nastou KC, Lyon D, Kirsch R, Pyysalo S (2021). The STRING database in 2021: customizable protein-protein networks, and functional characterization of user-uploaded gene/measurement sets. Nucleic Acids Res.

[25] Ru B, Wong CN, Tong Y, Zhong JY, Zhong SSW, Wu WC (2019). TISIDB: an integrated repository portal for tumor-immune system interactions. Bioinformatics.

[26] Xie B, Wang S, Jiang N, Li JJ (2019). Cyclin B1/CDK1-regulated mitochondrial bioenergetics in cell cycle progression and tumor resistance. Cancer Lett.

[27] Wu J, Lu LY, Yu X (2010). The role of BRCA1 in DNA damage response. Protein Cell.

[28] Yao G, Chen K, Qin Y, Niu Y, Zhang X, Xu S (2019). Long non-coding RNA JHDM1D-AS1 interacts with DHX15 protein to enhance non-small-cell lung cancer growth and metastasis. Mol Ther Nucleic Acids.

[29] Bray F, Jemal A, Grey N, Ferlay J, Forman D (2012). Global cancer transitions according to the human development index (2008–2030): a population-based study. Lancet Oncol.

[30] Frezza C (2020). Metabolism and cancer: the future is now. Br J Cancer.

[31] Yoshikumi Y, Mashima H, Ueda N, Ohno H, Suzuki J, Tanaka S (2005). Roles of CTPL/Sfxn3 and Sfxn family members in pancreatic islet. J Cell Biochem.

[32] Owada-Ozaki Y, Muto S, Takagi H, Inoue T, Watanabe Y, Fukuhara M (2018). Prognostic impact of tumor mutation burden in patients with completely resected Non-small cell lung cancer: brief report. J Thorac Oncol.

[33] Whitehall JC, Greaves LC (2020). Aberrant mitochondrial function in ageing and cancer. Biogerontology.

[34] Fleming MD, Campagna DR, Haslett JN, Trenor CC, Andrews NC (2001). A mutation in a mitochondrial transmembrane protein is responsible for the pleiotropic hematological and skeletal phenotype of flexed-tail (f/f) mice. Genes Dev.

[35] Tang M, Huang Z, Luo X, Liu M, Wang L, Qi Z (2019). Ferritinophagy activation and sideroflexin1-dependent mitochondria iron overload is involved in apelin-13-induced cardiomyocytes hypertrophy. Free Radic Biol Med.

[36] Sousa L, Garcia IJ, Costa TG, Silva LN, Renó CO, Oliveira ES, et al. Effects of Iron overload on the activity of Na,K-ATPase and lipid profile of the human erythrocyte membrane. PLoS One. 2015;10(7):e0132852. 10.1371/journal.pone.0132852 PMC4510300 26197432

[37] Seth Nanda C, Venkateswaran SV, Patani N, Yuneva M (2020). Defining a metabolic landscape of tumours: genome meets metabolism. Br J Cancer.

[38] Li S, Kuang M, Chen L, Li Y, Liu S, Du H, et al. The mitochondrial protein ERAL1 suppresses RNA virus Infection by facilitating RIG-I-like receptor signaling. Cell Rep. 2021;34(3):108631. 10.1016/j.celrep.2020.108631 33472079

[39] Breda CNS, Davanzo GG, Basso PJ, Saraiva Câmara NO, Moraes-Vieira PMM (2019). Mitochondria as central hub of the immune system. Redox Biol.

[40] Locasale JW (2013). Serine, glycine and one-carbon units: cancer metabolism in full circle. Nat Rev Cancer.

[41] Muthusamy T, Cordes T, Handzlik MAO, You L, Lim EW, Gengatharan J (2020). Serine restriction alters sphingolipid diversity to constrain tumour growth. Nature.

[42] Ribatti D, Ranieri G (2015). Tryptase, a novel angiogenic factor stored in mast cell granules. Exp Cell Res.

[43] Li F, Du X, Lan F, Li N, Zhang C, Zhu C, et al. Eosinophilic inflammation promotes CCL6-dependent metastatic tumor growth. Sci Adv. 2021;7(22): eabb5943. 10.1126/sciadv.abb5943 PMC8153717 34039594

[44] Pardoll DM (2012). The blockade of immune checkpoints in cancer immunotherapy. Nat Rev Cancer.

[45] Théate I, van Baren N, Pilotte L, Moulin P, Larrieu P, Renauld JC (2015). Extensive profiling of the expression of the indoleamine 2,3-dioxygenase 1 protein in normal and tumoral human tissues. Cancer Immunol Res.

[46] Prendergast GC, Mondal A, Dey S, Laury-Kleintop LD, Muller AJ (2018). Inflammatory reprogramming with IDO1 inhibitors: turning immunologically unresponsive ‘Cold’’tumors ‘Hot’. Trends Cancer.

[47] Tang K, Wu YH, Song Y, Yu B (2021). Indoleamine 2,3-dioxygenase 1 (IDO1) inhibitors in clinical trials for cancer immunotherapy. J Hematol Oncol.

[48] Chen W (2011). IDO: more than an enzyme. Nat Immunol.

