# Supplementary material for: Comprehensive analysis the prognostic and immune characteristics of mitochondrial transport-related gene SFXN1 in lung adenocarcinoma
Source: BMC Cancer. 2024 Jan 17;24:94. doi: 10.1186/s12885-023-11646-z (PMC10795352; doi:10.1186/s12885-023-11646-z)

**Supplementary figures**


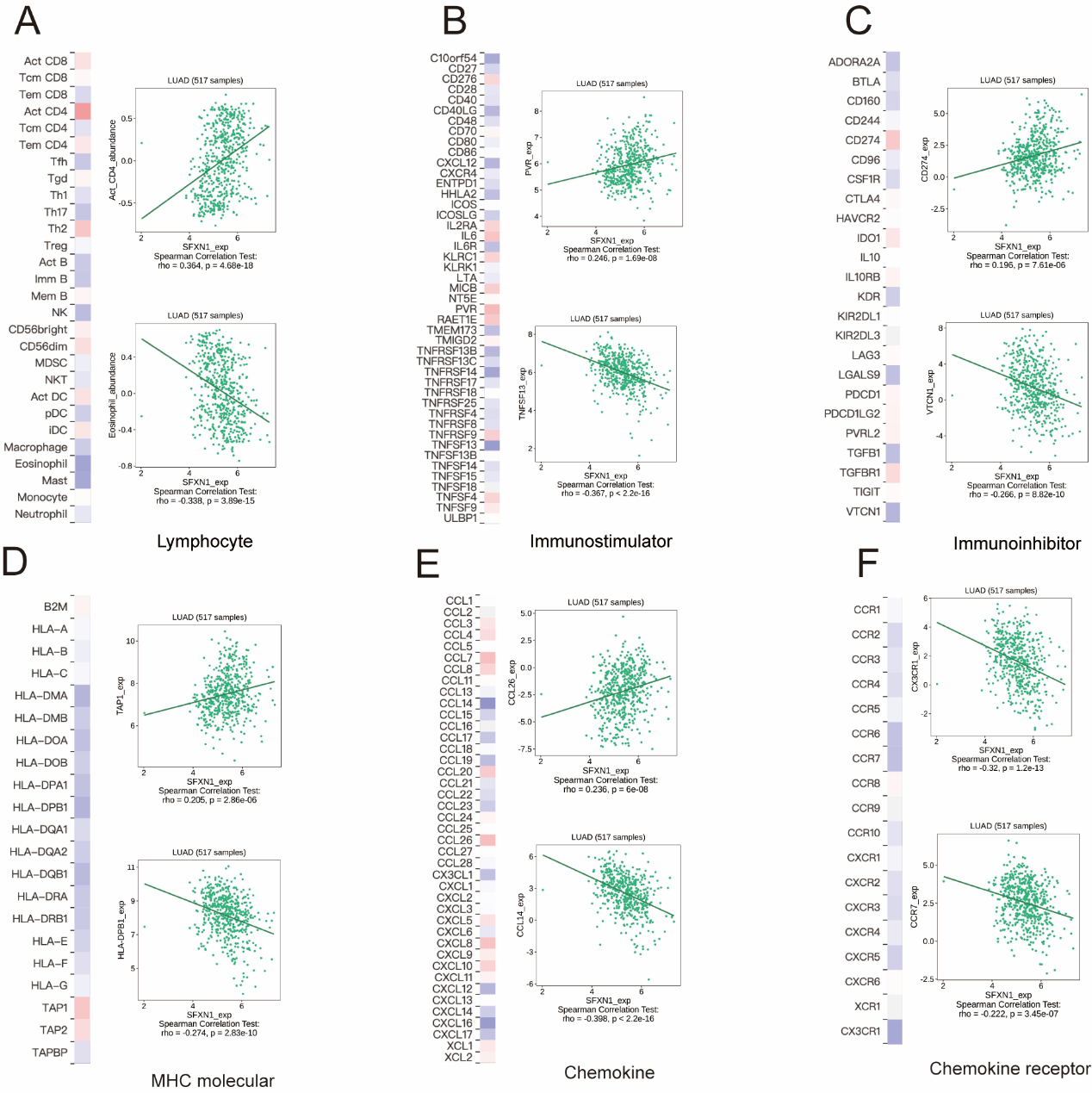


**Fig.S1 Association of SFXN1 expression with lymphocytes, immunomodulators, MHC molecules, chemokines and chemokine receptors**

**A-F** Correlation of SFXN1with lymphocytes(**A**), immunostimulators(**B**), immunoinhibitors(**C**), MHC molecules(**D**), chemokines (**E**) and chemokine receptors(**F**).


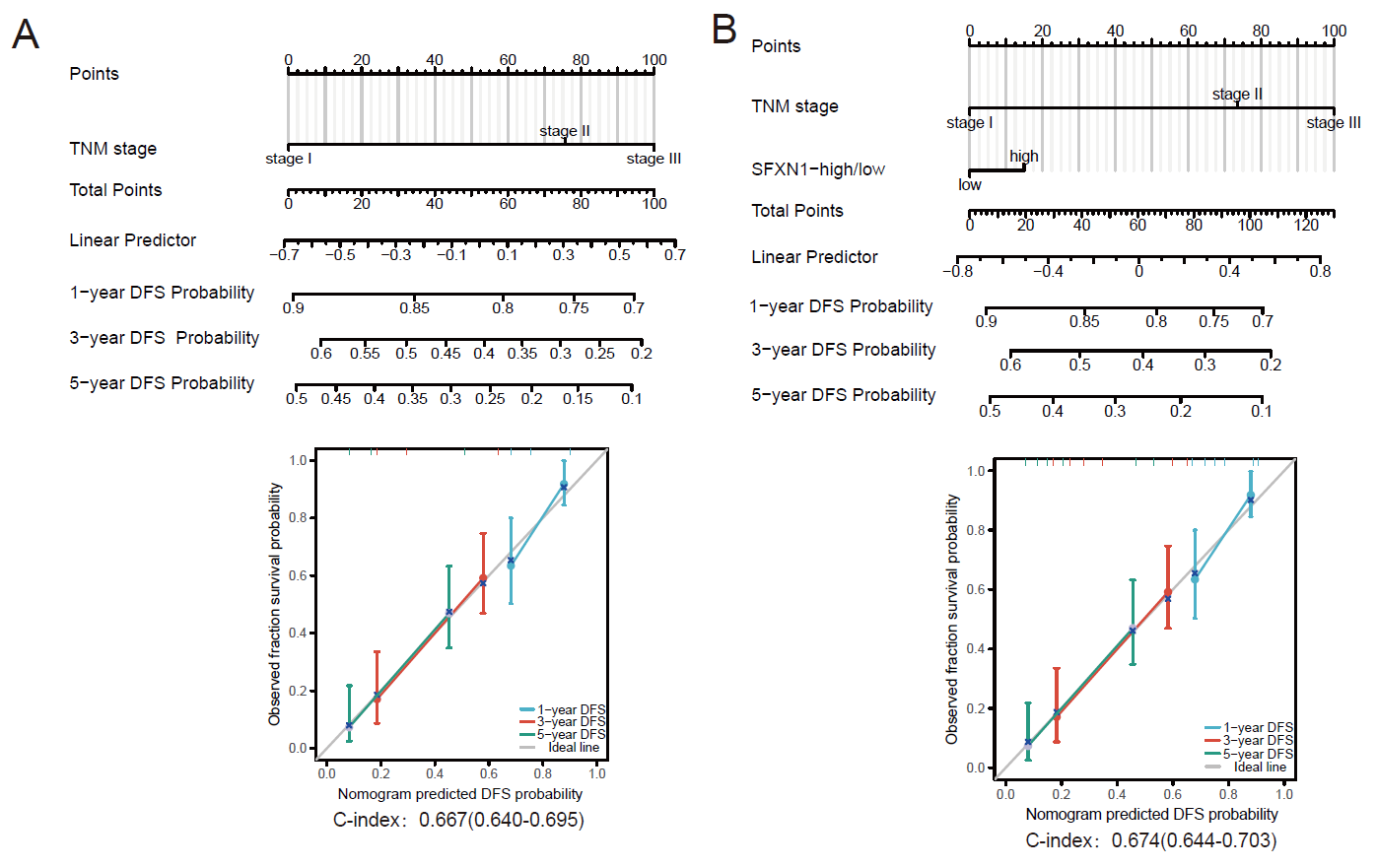


**Fig. S2** **Nomogram based on SFXN1 IHC score and TNM stage for DFS in validation set.**

**A** The nomogram and calibration curve based on TNM stage for DFS in LUAD. **B** The nomogram and calibration curve based on SFXN1 IHC score and TNM stage for DFS in LUAD.

**Supplementary tables**

**Supplementary Table 1** Other primes used in this study are as follows.

| Gene | Forward | Reverse |
| --- | --- | --- |
| CHEK1 | 5’-GTTCTGGCTGAGAACTGGAGTAC-3’ | 5’-GTTCTGGCTGAGAACTGGAGTAC-3’ |
| CHEK2 | 5’-GACCAAGAACCTGAGGAGCCTA-3’ | 5’-GGATCAGATGACAGCAGGAGTTC-3’ |
| ATR | 5’-GGAGATTTCCTGAGCATGTTCGG-3’ | 5’-GGCTTCTTTACTCCAGACCAATC-3’ |
| ATM | 5’-TGTTCCAGGACACGAAGGGAGA-3’ | 5’-CAGGGTTCTCAGCACTATGGGA-3’ |
| BRCA1 | 5’-CTGAAGACTGCTCAGGGCTATC-3’ | 5’-AGGGTAGCTGTTAGAAGGCTGG-3’ |
| BRCA2 | 5’-GGCTTCAAAAAGCACTCCAGATG-3’ | 5’-GGATTCTGTATCTCTTGACGTTCC-3’ |
| CDK1 | 5’-GGAAACCAGGAAGCCTAGCATC-3’ | 5’-GGATGATTCAGTGCCATTTTGCC-3’ |
| CDK4 | 5’-CCATCAGCACAGTTCGTGAGGT-3’ | 5’-TCAGTTCGGGATGTGGCACAGA-3’ |
| CDK6 | 5’-GGATAAAGTTCCAGAGCCTGGAG-3’ | 5’-GCGATGCACTACTCGGTGTGAA-3’ |
| CCNB1 | 5’-GACCTGTGTCAGGCTTTCTCTG-3’ | 5’-GGTATTTTGGTCTGACTGCTTGC-3’ |
| CCNA2 | 5’-CTCTACACAGTCACGGGACAAAG-3’ | 5’-CTGTGGTGCTTTGAGGTAGGTC-3’ |
| CCND1 | 5’-TCTACACCGACAACTCCATCCG-3’ | 5’-TCTGGCATTTTGGAGAGGAAGTG-3’ |
| RAD50 | 5’-GGAAGAGCAGTTGTCCAGTTACG-3’ | 5’-GAGTAAACTGCTGTGGCTCCAG-3’ |
| RAD51 | 5’-TCTCTGGCAGTGATGTCCTGGA-3’ | 5’-TAAAGGGCGGTGGCACTGTCTA-3’ |
| WEE1 | 5’-GATGTGCGACAGACTCCTCAAG-3’ | 5’-CTGGCTTCCATGTCTTCACCAC-3’ |
| MRE11 | 5’-CAGCAACCAACAAAGGAAGAGGC-3’ | 5’-GAGTTCCTGCTACGGGTAGAAG-3’ |
| PARP1 | 5’-CCAAGCCAGTTCAGGACCTCAT-3’ | 5’-GGATCTGCCTTTTGCTCAGCTTC-3’ |
| DHX15 | 5’-CACTGCTGAACGTCTACCATGC-3’ | 5’-CATTGTCTGCGGACATCAGGGA-3’ |

**Supplementary Table 2** The Cox regression analysis among clinical traits and DFS in validation set.

| **Characteristics** | **Total(N)** | **Univariate analysis** | |  | **Multivariate analysis** | |
| --- | --- | --- | --- | --- | --- | --- |
|  |  | **Hazard ratio (95% CI)** | **P value** |  | **Hazard ratio (95% CI)** | **P value** |
| **Gender** |  |  | 0.565 |  |  |  |
| Male | 47 | Reference |  |  |  |  |
| Female | 43 | 0.876 (0.557 - 1.376) | 0.565 |  |  |  |
| **Age** | 90 | 0.995 (0.970 - 1.020) | 0.686 |  |  |  |
| **Smoking history** |  |  | 0.884 |  |  |  |
| Non-smoker | 50 | Reference |  |  |  |  |
| Smoker | 40 | 1.034 (0.658 - 1.627) | 0.884 |  |  |  |
| **ECOG-PS** |  |  | 0.762 |  |  |  |
| 0 | 35 | Reference |  |  |  |  |
| 1 | 55 | 1.075 (0.673 - 1.715) | 0.762 |  |  |  |
| **TNM stage** |  |  | **< 0.001** |  |  |  |
| I | 41 | Reference |  |  | Reference |  |
| II | 8 | 2.689 (1.196 - 6.044) | **0.017** |  | 1.824 (0.495 - 6.722) | 0.367 |
| III | 41 | 3.700 (2.221 - 6.165) | **< 0.001** |  | 2.201 (0.670 - 7.229) | 0.193 |
| **T stage** |  |  | **< 0.001** |  |  |  |
| <3cm | 50 | Reference |  |  | Reference |  |
| ≥3cm | 40 | 3.478 (2.097 - 5.771) | **< 0.001** |  | 2.641 (1.515 - 4.604) | **< 0.001** |
| **N stage** |  |  | **< 0.001** |  |  |  |
| Negative | 43 | Reference |  |  | Reference |  |
| Positive | 47 | 3.217 (1.976 - 5.238) | **< 0.001** |  | 1.382 (0.439 - 4.345) | 0.580 |
| **IHC score of SFXN1** | 90 | 1.105 (1.002 - 1.219) | **0.045** |  | 0.947 (0.835 - 1.073) | 0.390 |

**WB original blots in supplementary information**

SFXN1 expression in LUAD cell lines and Beas-2B (**Figure 10B**)

1. **β-Actin(Replicate 1)**

**
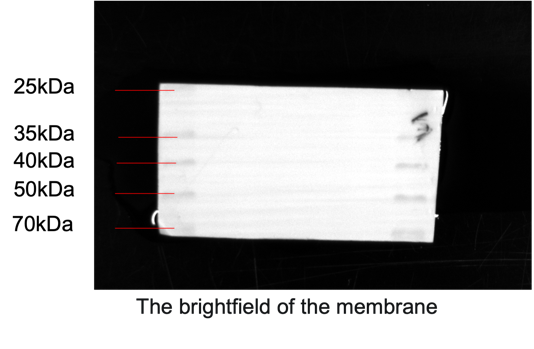
**
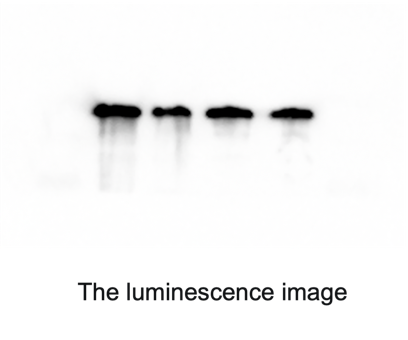


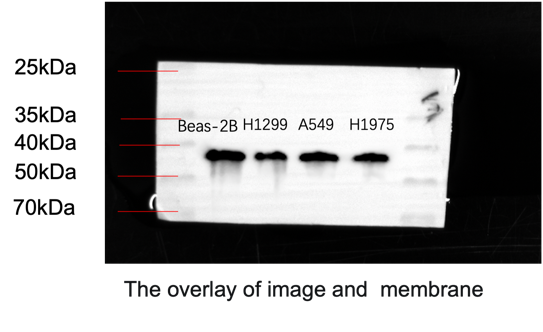


**SFXN1(Replicate 1)**

**
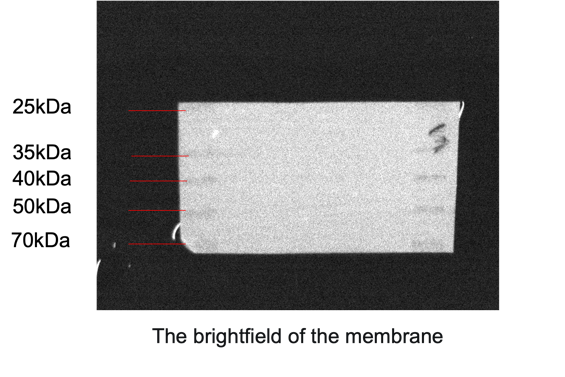

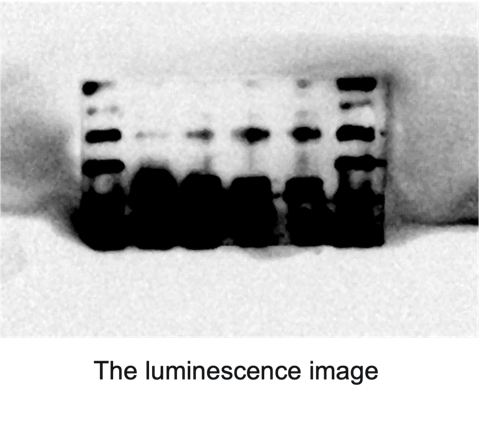
**

1. **β-Actin(Replicate 2)**

**SFXN1(Replicate 2)**

SFXN1 expression in A549 cell infected by control vector and overexpression vector (**Figure 4D**)

1. **β-Actin(Replicate 1) A549**

**SFXN1(Replicate 1) A549**

1. **β-Actin(Replicate 2) A549**

1. **SFXN1(Replicate 2) A549**

**Croppedblots in main paper (Figure 10B)**

**
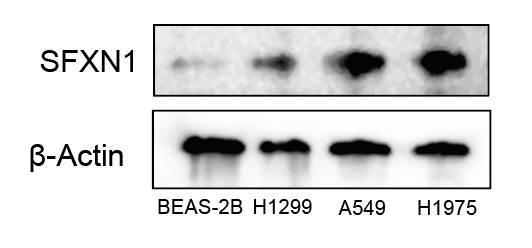
**

**Croppedblots in main paper (Figure 4D)**


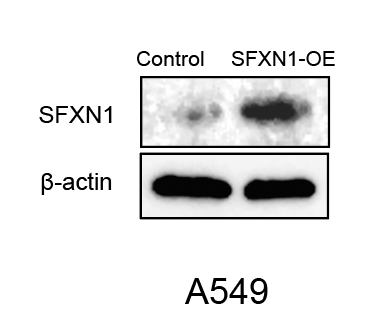

Supplement: Supplementary file 1 — Additional file 1: Supplementary figures, tables and WB original blots. Fig. S1. Association of SFXN1 expression with lymphocytes, immunomodulators, MHC molecules, chemokines and chemokine receptors. Fig. S2. Nomogram based on SFXN1 IHC score and TNM stage for DFS in validation set. Supplementary Table 1. Other primes used in this study are as follows. Supplementary Table 2. The Cox regression analysis among clinical traits and DFS in validation set. WB original blots in supplementary information. [file 12885_2023_11646_MOESM1_ESM.docx]
